# Investigating the Integration and the Long-Term Use of Smart Speakers in Older Adults’ Daily Practices: Qualitative Study

**DOI:** 10.2196/47472

**Published:** 2024-02-12

**Authors:** Fangyuan Chang, Lin Sheng, Zhenyu Gu

**Affiliations:** 1 Interaction Design Lab School of Design Shanghai Jiao Tong University Shanghai China

**Keywords:** smart speaker, private home, older adults, long-term use, daily practices, smart speakers

## Abstract

**Background:**

As smart speakers become more popular, there have been an increasing number of studies on how they may benefit older adults or how older adults perceive them. Despite the increasing ownership rates of smart speakers among older adults, studies that examine their integration and the long-term use in older adults’ daily practices are scarce.

**Objective:**

This study aims to uncover the integration of smart speakers into the daily practices of older adults over the long term, contributing to an in-depth understanding of maintained technology use among this demographic.

**Methods:**

To achieve these objectives, the study interviewed 20 older adults who had been using smart speakers for over 6 months. These semistructured interviews enabled participants to share their insights and experiences regarding the maintained use of smart speakers in the long term.

**Results:**

We identified 4 dimensions of the long-term use of smart speakers among older adults, including functional integration, spatial integration, cognitive integration, and semantic integration. For the functional integration of smart speakers, the study reported different types of use, including entertainment, information collection, medication reminders, companionship, environment modification, and emergency calls. For the spatial integration of smart speakers, the study showed older adults’ agency in defining, changing, and reshaping daily practices through the spatial organization of smart speakers. For the cognitive integration of smart speakers, the findings showed the cognitive processes involved in adapting to and incorporating smart speakers into daily habits and routines. For the semantic integration of smart speakers, the findings revealed that older adults’ enjoyable user experience and strong bonds with the device contributed to their acceptance of occasional functional errors. Finally, the study proposed several suggestions for designers and developers to better design smart speakers that promote maintainable use behaviors among older adults.

**Conclusions:**

On the basis of the findings, this study highlighted the importance of understanding how older adults use smart speakers and the practices through which they integrate them into their daily routines. The findings suggest that smart speakers can provide significant benefits for older adults, including increased convenience and improved quality of life. However, to promote maintainable use behaviors, designers and developers should consider more about the technology use contexts and the specific needs and preferences of older adults when designing these devices.

## Introduction

Along with the advancement of speech technology and artificial intelligence, smart speakers such as Google Home and Amazon Echo are becoming integral to households [1]. Equipped with smart voice assistants such as Google Assistant and Amazon Alexa, these devices respond to voice commands, facilitating activities like playing music, answering questions, setting reminders, and controlling smart home appliances. Their speech input and output features enhance accessibility, especially for individuals with limited mobility and vision [2,3].

In the realm of health care, the adoption of smart speakers has opened avenues for significant advancements, akin to the transformative impact of mobile phones. These devices present unique advantages for health research in out-of-hospital environments, offering opportunities for chronic disease management, passive identification of medical emergencies, detection of behavioral and cognitive changes, and remote monitoring of respiratory diseases impacting public health [4,5]. A range of successful pilot studies has demonstrated the positive impact of smart speakers on users requiring social care, and adults with learning differences, showcasing potential cost savings and improved well-being [6,7].

Despite the potential benefits, scholars have highlighted a notable gap in knowledge concerning older adults’ experiences with smart speakers in long-term use, which poses challenges to assessing the long-term impact and implications of these devices on the well-being and quality of life of older adults. Specifically, while research has explored smart speaker use among various demographics, such as low-income populations [8,9], people with disabilities [10,11], parents [12,13], and children [14], studies specifically focusing on the older adult demographic remain relatively scarce [3,15]. Among the limited studies, the majority of the existing literature concentrates on the design of smart speakers for later life, encompassing discussions on effective conversational cues [16], privacy concerns [17], and the anthropomorphism of speakers [18]. Other studies have assessed the feasibility and usability of smart speakers for promoting active aging [19,20], investigated older adults’ first impressions of smart speakers [19,21], and identified influential factors regarding older adults’ attitudes toward smart speakers [22]. The most recent studies emphasized there is a missing knowledge about older adults’ experiences with smart speakers in long-term use. This knowledge gap hinders the development of tailored interventions and policies to maximize the benefits of smart speakers for this demographic, ensuring their inclusion in the digital revolution and promoting healthy aging in the digital era [23-25]. Consequently, there is a pressing need for comprehensive exploration into older adults’ long-term experiences with smart speakers, emphasizing their perceptions, attitudes, and the evolving nature of their interactions with this technology.

This study aims to understand how older adults routinely use smart speakers and integrate them into their daily lives. The outcomes can add to the emerging body of literature for a more comprehensive understanding of older adults’ use of smart speakers in the long term. We conducted semistructured interviews with 20 older adults who used the smart speaker for over half a year, to answer the following research questions: “How does the use of smart speakers integrate into older adults’ daily practices?” and “What design considerations can be generated from the long-term use of smart speakers by older adults?”

In summary, our study embarks on an exploration into the long-term experiences of older adults with smart speakers, filling a notable gap in the existing knowledge. While previous research has delved into aspects such as conversational cues, the feasibility of smart speakers for active aging, and the before-and-after adoption changes or the broader impact, the focus on the nuanced, day-to-day interactions of older adults with these devices over an extended period is a novel dimension that sets our study apart [26]. By unraveling the complexities and diverse experiences during older adults’ long-term engagement with smart speaker technology, we aim to contribute not only to the effective design of age-friendly devices but also to the broader discourse on the role of technology in promoting well-being among older populations.

## Methods

### Study Design

A qualitative research study was conducted to investigate the experiences of older adults’ long-term use of smart speakers in daily practices. Semistructured interviews were used to facilitate an in-depth exploration of older adults’ experiences, even when the study involved a relatively small number of participants [27,28]. The study adhered to the guidelines and reporting standards outlined in the Consolidated Criteria for Reporting Qualitative Research (COREQ) checklist for qualitative studies [29].

### Ethical Considerations

Ethical approval for the research was obtained from the research ethics committee of the Shanghai Jiao Tong University (H2022335I). Informed consent was obtained from all participants.

### Participants and Recruitment

The research was conducted in Shanghai, China. To ensure diverse perspectives, participants were recruited from 2 distinct communities of older people—one situated in a bustling urban environment and the other in a rural area on the outskirts of the city. In the urban community, the researchers partnered with a community coordinator who had established relationships with older adults. In the rural community, the research team collaborated with the head of the older people’s community, who was responsible for arranging community activities. The coordinator and the community head served as a liaison between the research team and the two communities to help in identifying suitable candidates.

The specific criteria for participant inclusion in the study remained consistent across both communities, requiring individuals to be aged 60 years or older and to have a minimum of 6 months of experience using a smart speaker in their homes. The time limit served to ensure that current practices of using smart speakers were represented and to improve the reliability of experience recollection. Therefore, to be included in the study, participants needed to be aged 60 years or older and have had at least 6 months of experience using a smart speaker in their homes. Community members were thus excluded if they did not meet the age requirement, or had less than 6 months of experience with a smart speaker.

During the recruitment process, the research team first contacted the community coordinator or the community head, who shared the information about this research with all the members of the community. The older adults who met the inclusion criteria and showed their interest in participating in the study were recruited. The snowball approach was then used to identify more older adults using smart speakers for more than 6 months. Those who met the inclusion criteria were recruited as participants. The participants were compensated with gift cards (valued at around US $9) for interview participation. Ultimately, 20 older adults were recruited (Table 1). The sample size is in line with the recommendations by Guest et al [30] and Hennink et al [31], who pointed out that empirical data reached saturation within 20 interviews. Other qualitative literature about older adults’ use of technology used a similar number of participants [24,32].

**Table 1:** Demographics of participants.

Label	Sex	Age (years)	Years of experience^a^	Technical proficiency^b^	Used smart speaker
OA1^c^	Female	72	2	Intermediate	Brand A
OA2	Female	66	0.5	Intermediate	Brand A
OA3	Female	69	1	Novice	Brand A
OA4	Female	71	1	Novice	Brand A
OA5	Male	73	3	Novice	Brand B
OA6	Male	67	2.5	Novice	Brand B
OA7	Male	68	0.5	Intermediate	Brand A
OA8	Female	61	0.5	Novice	Brand B
OA9	Female	73	2	Intermediate	Brand A
OA10	Female	66	2	Novice	Brand A
OA11	Male	77	1.5	Intermediate	Brand B
OA12	Female	65	2	Novice	Brand B
OA13	Male	73	1.5	Intermediate	Brand B
OA14	Female	64	1	Novice	Brand A
OA15	Female	73	1.5	Novice	Brand A
OA16	Male	73	1	Intermediate	Brand B
OA17	Male	62	0.5	Intermediate	Brand B
OA18	Male	64	1	Novice	Brand B
OA19	Female	69	2	Novice	Brand A
OA20	Female	70	1.5	Intermediate	Brand B

^a^The actual time that the participant starts to use the smart speaker regularly (self-reported).

^b^Individuals’ ability to discern the usefulness of a smart speaker and their comfort in using it for various purposes (evaluated by the Artificial Intelligence Literacy Questionnaire proposed in [33], Multimedia Appendix 1).

^c^OA: older adult.

### Data Collection

A literature review of technology use among older adults informed the development of a flexible and semistructured interview guide. The interview guide was developed in consultation with a professor with expertise in qualitative data collection and analysis. One preliminary interview was conducted with the included community coordinator (aged 58 years) to ensure the fluency of formal interviews (eg, whether questions are easy to understand). As the goal of our study was to better understand older adults’ long-term use of smart speakers in home environments, the research questions were developed encompassing their practices, experiences, values, and expectations about using the smart speaker (Multimedia Appendix 2).

All interviews received written consent and were conducted in a private confidential environment such as the community activity room and private homes between November 2022 and September 2023. The participants were encouraged to give examples to contextualize their daily practices of using the smart speaker. The interviews ranged in length between 30 and 60 minutes. Participants who had limited knowledge or experience in using smart speakers had shorter interviews. All interviews were audio-recorded. During the interview, we conducted regular summaries to ensure the validity of the collected data.

### Data Analysis

All interviews were transcribed. The interview transcripts were reported in a way to ensure the anonymity of the participants. The collected data were interpreted by inductive content analysis [34], with a focus on how older adults use the smart speaker in their home environments, and why the speaker is used in the observed way. The transcripts were analyzed in NVivo (version 12; Lumivero) following the process proposed by Braun and Clarke [27]. After a data familiarization stage, 2 authors (FC and LS) separately extracted text segments related to the research goal and categorized them into codes. Applying an iterative process, the relationships between codes were analyzed and the subthemes were formed by grouping related segments together. The 2 authors compared the codes and subthemes and resolved any differences in coding through discussion. The assignment of text segments to the subcategories was repeatedly checked to see if they reflected the same meaning. Data saturation was determined when no new themes and relationships among the interview data were found [35]. As a result, a list of 4 themes and 12 subthemes, upon which the authors achieved an agreement, was generated [36].

## Results

### Overview

We identified 4 dimensions of the long-term use of smart speakers among older adults, including functional integration, spatial integration, cognitive integration, and semantic integration (Table 2).

**Table 2:** Identified themes, subthemes, and code examples related to the long-term use of smart speakers among older adults.

Themes and subthemes		Code examples
**Functional integration**		
	Entertainment	Using it for listening to music Chatting with the small thing for fun Telling some stories
	Information collection	Hear daily news Weather information A useful tool for knowing what is going on
	Medication reminders	The speaker can send out medication reminders When I see it, I know, “Oh I need to take pills”
	Companionship	Enjoy hearing the speaker’s answers The speaker is accompanying me, he is a friend Love sitting in the coach, with her beside me
	Environment modification	Just need to say “Turn off the lights” Connect it with the switch Connect to the camera to show who is knocking on the door
	Emergency calls	Call for help if falling on the ground
**Spatial integration**		
	Smart speaker location	Decide the device’s location according to daily habits Put it beside the medicine box for task convenience
	Appliance connection	Locate the smart speaker in the bedroom for device connection Put the smart speaker beside the door for better internet Put it on the bedside table for easily hearing the doorbells
**Cognitive integration**		
	Learning and problem-solving	Memorize the required voice commands Handle the emerging technical hurdles
	Mental adaptation	Overcome skepticism and building trust Realize the benefits of smart speakers for personal convenience
**Semantic integration**		
	Enjoyable experience	Save energy in memorizing things Assist older adults in performing multitask operations
	Affective bonds	Feel a shared and caring presence through the technology use Experience tranquility while reading with the smart speaker

### Functional Integration of Smart Speakers

Functional integration relates to the ways in which older adults use smart speakers to meet various functional needs in their daily lives. This dimension focuses on the practical and functional aspects of smart speaker use and how they are integrated into daily practices. Our findings revealed the diversity of use genres that had been developed in older adults’ use of smart speakers in their living environments. Though most participants were using a smart speaker of the same brand, the ways of using the device were multiple, including entertainment, information collection, medication reminders, companionship, environment modification, as well as emergency calls.

The most commonly mentioned use genre was entertainment. Almost all participants said they use the smart speaker to listen to music.

...I often listen to songs [via the smart speaker]. Some songs could be used for our square dance.

Female, age 69 years

In addition to listening to music, 1 participant mentioned that he once used the smart speaker to recognize the name of a song.

...I was recalling the name of a song. My granddaughter told me if I sang the song’s melody, the speaker could tell me the song’s name.

Male, age 73 years

Similar to the example above, using the smart speaker for information collection was widely found in the interviews. Our participants used the smart speaker to gain information such as daily weather, local news, and safety tips related to the COVID-19 pandemic.

The smart speaker was also commonly used for medication reminders. The participants indicated that they had to always remember when to take medicines before using the smart speaker. With the help of their families or relatives, they set up the smart speaker to remind them to take medicines at a specific time.

...I have to take medicines every day.... Now the speaker can remind me of taking medicines.

Female, age 66 years

Besides, the participants emphasized that they benefited from the companionship of the device. The following example showed how older adults saw the smart speaker as a person who accompanied them, and enjoyed the conversation with the smart speaker.

...My smart speaker is a well-behaved little girl, just like my granddaughter. She wakes me up every morning. I say good morning to her sometimes. Sometimes I ask her some questions that I know the answers.... I am just curious about how she would reply to me, and enjoy hearing her answers.

Male, age 73 years

Another use genre was about environment modification. A few participants used the smart speaker to control other smart appliances in their living environments. For instance, 1 participant explained that the smart speaker helped overcome the inconvenience caused by switches that were in distant places.

The switch in my bedroom is beside the door.... I had to get out of bed, switch off the light, and then get into the bed in the darkness.... Now I just need to say “turn off the lights.”

Female, age 72 years

Finally, 1 participant mentioned that she had not used the smart speaker for emergency calls, but she knew she could call her daughters through the smart speaker or a “white button” when necessary.

I asked the smart speaker to call my daughter, her phone rang. I clicked the white button, her phone rang...[a white button, about 5 cm in diameter, is shown in her hand].

Female, age 61 years

### Spatial Integration of Smart Speakers

Spatial integration relates to the ways in which older adults use the physical space and placement of their smart speakers to shape and organize their daily routines. This dimension focuses on the physical aspects of smart speaker use. Our findings suggested that participants located their smart speakers in different living spaces, including the living room, the bedroom, the study, the bathroom, and the balcony. By spatially organizing the speaker, and connecting it with other smart appliances or daily tasks in these contexts, the participants built up the use contexts under which they are comfortable interacting with the smart speakers, and actively changed and reshaped their daily practices.

The following example showed how participants decided the locations of the smart speaker and formed new daily practices according to the purposes for using the smart speaker and the existing daily practices.

...I wanted to take the pills, I had to get water from the kitchen, so I put the speaker, together with the medicine box, in the corner of my kitchen now.

Female, age 73 years

Some participants incorporated the smart speaker by connecting it with the existing appliances in their living environments. In doing so, their practices of completing specific tasks were simplified.

...The light in my bedroom can be connected and controlled by the smart speaker, I thus put it in my bedroom. Now I just need to say “turn off the lights” when I want to sleep.

Female, age 72 years

### Cognitive Integration of Smart Speakers

Cognitive integration relates to the way in which older adults adapt to and incorporate smart speakers into daily habits and routines from a cognitive perspective. Participants shared their experiences of familiarizing themselves with the smart speaker’s user interface and functionalities. The impact of successful problem-solving was evident in the participants’ narratives. Many acknowledged that navigating through and resolving issues not only enhanced their understanding of the device but also strengthened their overall intention to use the smart speaker in the long term. Overcoming learning or technical challenges contributed to a sense of accomplishment and increased confidence in using the smart speaker.

Almost all participants mentioned the challenges in learning voice commands and the overall functionality of the device, as well as resolving technical hurdles. They also mentioned the importance of adept problem-solving skills when facing these challenges. Some participants recounted instances of encountering glitches such as software malfunctions, intermittent connectivity issues, and occasional misinterpretations of voice commands by the smart speaker. One participant shared a specific experience, recalling:

There were times when it simply didn’t respond, leaving me puzzled. I had to delve into troubleshooting methods to identify the issue.

Female, age 64 years

Another participant highlighted the initial difficulty in grasping the full range of functions, expressing:

Mastering the voice commands posed a bit of a challenge. Instead of saying “XX, I need...” you have to initiate with “Hi, XX.”...took me some time to unravel the full range of functions it offered.

Male, age 62 years

In response to these challenges, older adults demonstrated resilience and resourcefulness in addressing technical issues. Problem-solving strategies ranged from seeking help from family members or friends to experimenting with different emotional and cognitive aspects of smart speaker use. Some engaged in hands-on exploration, experimenting with different commands to enhance their functional and mental understanding. Additionally, participants discussed seeking guidance from families, internet-based resources, and user manuals to expedite the adaptation process. As 1 participant shared:

I watched some tutorial videos online and read the manual, and asked my family. It helped me get a better grasp of what the smart speaker could do.

Female, age 65 years

As smart speakers became integrated into daily routines, participants described making gradual adjustments. The learning curve transformed into a journey of mental adaptation, with older adults incorporating the device into activities such as setting reminders, checking the weather, or even incorporating it into their medication routines. One participant shared the experience of overcoming skepticism and building trust with the smart speaker over time.

I was skeptical at first...But as I gradually used it, I found myself relying on it more.

Male, age 73 years

The transformative power of smart speakers was evident in their ability to enhance social interactions during family gatherings. One user realized the potential of the device in this context, stating:

I realized the potential of using the smart speaker during family gatherings. Now, we use it to play music, share interesting facts, and even settle debates.

Male, age 64 years

Similarly, another user’s experience highlighted the versatile nature of the smart speaker. Initially used for simple tasks like checking the weather and setting alarms, the device became a gateway to a multitude of possibilities.

Initially, I used it mainly for weather updates and setting alarms. But then, I discovered I could ask it to read audiobooks or provide cooking tips. It’s like unlocking a treasure trove of possibilities.

Female, age 70 years

### Semantic Integration of Smart Speakers

Semantic integration refers to the emotional and meaningful connections that older adults develop with their smart speakers over time. This dimension focuses on the emotional and cognitive aspects of smart speaker use. According to our data, although the smart speakers had occasional functional errors that annoyed the participants, all participants acknowledged, appreciated, and valued the benefits offered by using the smart speakers. The benefits included not only enjoyable experiences to complete daily tasks but also bonds with the device from an affective dimension. The enjoyable user experiences and the strong bonds with the smart speaker enhanced the participants’ positive attitudes toward their device, making them feel that occasional functional errors were acceptable.

The participants claimed that the smart speaker enabled them enjoyable experiences such as helping them access more information and enjoy more forms of entertainment. Besides, some said the speaker empowered them to multitask and saved time and energy. Yet, looking solely at the daily tasks completed by smart speakers, participants thought the smart speakers were not unreplaceable. As 1 participant indicated:

Apparently the smart speaker makes the completion of some daily tasks easier...but if only looking at the tasks it supports, I feel it doesn’t have that much uniqueness.

Female, age 69 years

The same person, however, emphasized that her affective bonds with the smart speaker meant a lot to her.

...what I feel uniqueness is the companionship offered by the smart speaker...by using it, I feel as if someone is experiencing every day together with me, and taking care of me.

Female, age 69 years

Other participants also emphasized the importance of companionship offered by the smart speaker. Some stated that they started to develop bonds with the smart speaker because of its capability for task completion, but the bonds were strengthened and maintained through the companionship over time, driving them to maintain their use behaviors.

...My son bought the smart speaker for me, because my friend in the community said it was convenient to listen to music.... Gradually [the speaker] becomes a “person” accompanying me.... I feel peace of mind when I am reading a book while he is just beside me, in the room together with me.

Male, age 67 years

The functional errors that emerged during the technology use were spontaneously mentioned by our participants. However, people looked at the errors with charity. Some even treated these errors as fun. For example, 1 participant mentioned that sometimes the smart speaker may be activated by the television sound.

Once, the leading actor said “I don’t love you,” the smart speaker replied “That is heartbreaking” [laughing].... I like it, I think these so-called “functional errors” are acceptable, but sometimes are also fun.

Male, age 67 years

In this case, the smart speaker was not viewed as a pure device whose fluency in function operations was prioritized. Instead, the participant treated the smart speaker as a social agent as its inappropriate responses activated by television was unexpected social actions (ie, conversations between different devices).

## Discussion

### Principal Findings

The study aimed to investigate how smart speakers are integrated into the daily practices of older adults in the long term. The findings of our study illuminate a nuanced understanding of how older adults seamlessly integrate smart speakers into their lives, encompassing functional, spatial, cognitive, and semantic dimensions. This integration aligns with existing literature, shedding light on the multifaceted benefits and challenges associated with the adoption of smart speaker technology among older populations.

### Integrate Multiple: Functional, Spatial, Cognitive, and Semantic Technology Integration

The functional integration of smart speakers among older adults is characterized by a rich diversity of use genres, reflecting the adaptability of these devices to meet various needs. Entertainment emerged as a dominant use genre, with participants expressing a universal affinity for using smart speakers to listen to music, transforming the device into a musical companion for activities like square dancing. This aligns with previous studies emphasizing the role of smart speakers in enhancing leisure activities for older adults [37]. Beyond entertainment, the device’s pivotal role in information collection, medication reminders, and environment modification resonates with literature, highlighting smart speakers as valuable tools for health management and home automation among older adults [38]. The potential for emergency calls, while not frequently used, aligns with the findings of studies emphasizing the importance of safety features for older adults [39]. The acceptance of smart speakers as reliable companions for various tasks aligns with the idea that these devices can address specific needs and preferences, contributing to the overall well-being of older users [40].

Spatial integration revealed how older adults strategically positioned smart speakers to shape and organize their daily routines. The participants allocated their speakers across diverse living spaces, from the living room to the bedroom, study, bathroom, and even the balcony. This spatial organization facilitated the creation of specific use contexts, where the smart speaker seamlessly blended into existing daily practices. This personalized approach aligns with literature emphasizing the importance of tailoring technology to older adults’ physical environments [41]. The integration of smart speakers with daily routines, such as pill-taking in the kitchen or controlling bedroom lighting, showcases the adaptability of older adults in incorporating technology seamlessly into their daily lives, emphasizing the active role older adults play in shaping their living environments to accommodate and optimize the technology functionality [42,43].

Cognitive integration illuminated the adaptive processes through which older adults familiarized themselves with the smart speaker’s functionalities and overcame learning challenges. The initial hurdles in mastering voice commands and comprehending the device’s full range of functions were common experiences. Yet, the participants’ narratives highlight their resilience and problem-solving acumen, aligning with studies that stress the importance of user support and educational resources in facilitating older adults’ technology adoption [44]. The gradual adjustments and incorporation of the smart speaker into daily habits reinforce the idea that cognitive integration is an ongoing process, transforming initial challenges into a journey of mental adaptation [45].

Semantic integration delved into the emotional and meaningful connections older adults forged with their smart speakers. Despite occasional functional errors, participants universally acknowledged and valued the benefits offered by these devices. The smart speaker’s role in facilitating enjoyable experiences, multitasking, and saving time and energy contributed to positive attitudes among older adults. The findings speak against the common discourse that technical issues and occasional functional errors could significantly diminish the overall positive impact and perceived value of smart speakers among older adults [46]. Instead, participants not only accepted these imperfections with understanding but also found moments of humor and enjoyment in the device’s occasional quirks. This resilience toward technical glitches underscores the robust emotional and meaningful connections formed between older adults and their smart speakers [24,47], highlighting that the perceived benefits and companionship offered by the technology far outweigh occasional operational hiccups.

To sum up, the integration of smart speakers among older adults is a holistic process that involves functional use, spatial organization, cognitive adaptation, and semantic connection with the device. Understanding the interplay of these dimensions provides a comprehensive insight into how smart speakers become integral components of older adults’ daily lives, offering not only practical functionalities but also emotional fulfillment and companionship.

### Design Implications

#### Being Aware of the Heterogeneity in Older Adults’ Technology Use

In our study, older adults used the smart speaker in different physical spaces, through different actions, and for different purposes. The underlying reasons could be the diversity of personalities [33], the differences in sociocultural and socioeconomic situations [48], and the disparities in individual digital literacy [49]. The heterogeneity in older adults’ technology use may pose challenges for designers and developers to transform ideas and insights into concrete designs. Merely focusing on a specific issue met by older adults would limit the benefits of smart speakers to older adults. The complex entanglement of space, practices, and user needs that may affect technology use at different sites should be carefully considered. We thus suggest designers and developers to zoom out and get the big picture of older adults’ daily practices before zooming in to define the design problem to solve.

#### Serving for the Existing Activities in Older Adults’ Home Environments

Despite the discourse that smart speakers can support older adults to explore unexperienced activities, in our study, participants used the device to enhance the quality of their existing activities. For instance, the smart speaker supports medication reminding, turning on or off the lights, and music listening. Consequently, we suggest designers and developers to pay more attention to the issues or opportunities related to older adults’ existing activities at home, rather than new activities that are not commonly experienced among this group of population.

#### Attaching Importance to Additional Devices Centered on the Smart Speaker

Our findings show that some additional devices connecting to the smart speakers were used by older adults. For instance, a “white button,” designed for emergency calls was carried by a participant all the time. The solution of connecting smart speakers to light switches in case of harm in darkness was also acknowledged. The 2 cases suggest that additional devices, especially the ones ensuring personal safety, have a market in the aging groups. Because many older adults have difficulties using the apps on mobile phones [3], we argue more additional devices centered on the smart speaker are needed.

#### Leaving Space for the Agency of Older Adults

Older adults are usually treated as inexpert technology users, and judged to be risky to do technology appropriation [50]. Yet, our findings show how participants spatially organized the smart speaker, and actively defined, changed, and reshaped their practices after using the device. In our cases, older adults created solutions that suit their specific needs [51]. These solutions were not predefined or preformatted by someone else, but specifically for older adults themselves [52]. We believe this may contribute to developing strong affective bonds between older adults and the device, driving their maintainable use behaviors, and promoting their resilient attitudes toward functional errors. Hence, we call for more studies about the association between older adults’ agency in technology use and their affective bonds with technology. We also encourage designers and developers to carefully consider the space for the agency of older adults in terms of technology appropriation.

#### Getting to Know the Preferences of Communities

The most commonly mentioned genre of using the smart speaker was listening to music in our study. This is understandable as many participants are from a square dancing community. It shows that the community where older adults are involved can implicitly manifest their preferences toward the purposes of technology use. Therefore, we encourage designers and developers to shift partial attention from individuals to communities when investigating the preferences of older adults.

As smart speakers swiftly reach the mainstream, understanding the detailed, nuanced experiences of older adults using these devices becomes paramount. Our research delves into the intricacies of daily use. By unraveling the complexities and diverse experiences during older adults’ long-term engagement with smart speaker technology, we aim to bridge the gap between technology and aging, offering insights that go beyond design considerations to impact policies and interventions. Moreover, our study seeks to contextualize the significance of understanding older adults’ experiences within the broader sociotechnological landscape. The potential benefits of these devices in health care, chronic disease management, and emergency identification align with broader societal challenges in health care accessibility and aging populations. In an age where digital advancements redefine societal norms, the integration of smart speakers into the lives of older adults is not just a technological trend; it is a pivotal factor in ensuring their inclusion in the digital revolution. Consequently, this research does not merely contribute to the attractiveness of smart speakers for older users but serves as a critical voice in the ongoing dialogue about healthy aging in the digital era.

### Conclusions

This paper focuses on the integration of smart speakers into older adults’ daily practices. We aim to understand how older adults routinely use smart speakers and integrate them into their daily lives, which in turn enables us to propose suggestions for designers and developers. A total of 20 older adults who have used a smart speaker for over 6 months were interviewed. The findings demonstrate that older adults integrate smart speakers into daily use through functional integration, spatial integration, cognitive integration, and semantic integration. Based on the findings, we proposed several suggestions for designers and developers to better design smart speakers that promote maintainable use behaviors by older adults.

### Limitations

Our findings must be evaluated within the context of several limitations. First, participants were recruited from 2 communities, potentially impacting the generalizability of the results. The risk of selection bias or unaccounted factors, such as the homogeneity of participant characteristics within the same facility, may have influenced interview responses. Yet, the study indeed highlighted the intricate nature of long-term technology use among older adults in these specific communities, offering valuable insights for designers and developers in the context of smart speaker design. Second, our study only investigated the long-term use of smart speakers, without paying much attention to the discontinuation of using smart speakers in the long term. The investigation of the discontinued use of smart speakers may reveal more insights into older adults’ technology use. However, we believe that it is important to examine the integration of smart speakers in older adults’ daily practices, as the findings may contribute to the maintainability of older adults’ technology use.

## Appendix

Multimedia Appendix 1 Artificial Intelligence Literacy Questionnaire (adapted).

Multimedia Appendix 2 Interview schedule.

## Abbreviations

COREQ: Consolidated Criteria for Reporting Qualitative Research
