# Supplementary material for: Investigating the Integration and the Long-Term Use of Smart Speakers in Older Adults’ Daily Practices: Qualitative Study
Source: JMIR Mhealth Uhealth. 2024 Feb 12;12:e47472. doi: 10.2196/47472 (PMC10897797; doi:10.2196/47472)
Supplement: Multimedia Appendix 1 [file mhealth_v12i1e47472_app1.docx]

| **Statement** |
| --- |
| I can distinguish between smart devices and non-smart devices.  I do not know how the smart speaker can help me.  I can identify the AI technology employed in the applications and products I use.  I can skillfully use the smart speaker to help me with my daily work.  It is usually hard for me to learn to use a new smart speaker.  I can use the smart speaker to improve my task efficiency.  I can evaluate the capabilities and limitations of a smart speaker after using it for a while.  I can choose a proper solution from various solutions provided by the smart speaker.  I can choose the most appropriate smart speakers from a variety for a particular task.  I always comply with ethical principles when using the smart speaker.  I am never alert to privacy and information security issues when using the smart speaker.  I am always alert to the abuse of smart speakers (privacy). |
