# Supplementary material for: Investigating the Integration and the Long-Term Use of Smart Speakers in Older Adults’ Daily Practices: Qualitative Study
Source: JMIR Mhealth Uhealth. 2024 Feb 12;12:e47472. doi: 10.2196/47472 (PMC10897797; doi:10.2196/47472)
Supplement: Multimedia Appendix 2 [file mhealth_v12i1e47472_app2.docx]

| **Focus area** | **Examples of questions and probes** |
| --- | --- |
| Daily practices | - Tell me a little about your day at home. How do you use the smart speaker?  - How was your day at home before having the smart speaker? |
| Interactions with the smart speaker | - How well do you think that you have been supported by using the speaker?  - Where do you put it? Why?  - Has there been an occasion when something you expected to happen by using the speaker didn’t happen? Tell me more about that. |
| Values of using the smart speaker | - In your own words, can you describe the relationship between you and the smart speaker?  - In comparison to other technology, what is the uniqueness of the smart speaker? |
